# Time trends in physical activity of adult users of the Brazilian National Health System: 2010-2014. Longitudinal study: Tendência temporal da atividade física de adultos usuários do sistema público de saúde brasileiro: 2010-2014. Estudo longitudinal

**DOI:** 10.1590/1516-3180.2017.0025190317

**Published:** 2017-08-07

**Authors:** Bruna Camilo Turi, Jamile Sanches Codogno, Rômulo Araújo Fernandes, Kyle Robinson Lynch, Eduardo Kokubun, Henrique Luiz Monteiro

**Affiliations:** I MSc, PhD. Researcher, Postgraduate Program on Kinesiology, Universidade Estadual Paulista Júlio de Mesquita Filho (UNESP), Rio Claro (SP), Brazil.; II MSc, PhD. Professor, Department of Physical Education, Universidade Estadual Paulista Júlio de Mesquita Filho (UNESP), Presidente Prudente (SP), Brazil.; III Master’s Student, Postgraduate Program on Kinesiology, Universidade Estadual Paulista Júlio de Mesquita Filho (UNESP-RC), Rio Claro (SP), Brazil.

**Keywords:** Motor activity, Primary health care, Public health, Risk factors, Epidemiology

## Abstract

**CONTEXT AND OBJECTIVE:**

In this longitudinal study, we aimed to describe time trends of physical activity (PA) in different domains from 2010 to 2014 among users of the Brazilian National Health System, taking into account the effects of sex, age and economic status (ES).

**DESIGN AND SETTING:**

Longitudinal study conducted in five primary care units in Bauru (SP), Brazil.

**METHODS:**

The sample was composed of 620 men and women who were interviewed in 2010, 2012 and 2014. The same group of researchers conducted the interviews, using the questionnaire developed by Baecke et al. Scores for occupational, exercise/sport, leisure-time/transportation and overall PA were considered in this longitudinal survey. Time trends of PA over the four years of follow-up were assessed according to sex, age and ES.

**RESULTS:**

We found that after four years of follow-up, the reduction in overall PA (-13.6%; 95% confidence interval, CI = -11.9 to -15.3) was statistically significant. Additionally, declines in the occupational domain and exercise/sports participation were affected by age, while the reduction in overall PA was affected by sex, age and ES.

**CONCLUSIONS:**

Overall PA decreased significantly from 2010 to 2014 among these outpatients of the Brazilian National Health System, and age and male sex were important determinants of PA in its different domains.

## RESUMO

**CONTEXTO E OBJETIVO:**

Neste estudo longitudinal, o objetivo foi descrever as tendências temporais de atividade física (AF) em diferentes domínios de 2010 a 2014 entre usuários do Sistema Único de Saúde, levando em conta o efeito do sexo, idade e condição econômica (CE).

**TIPO DE ESTUDO E LOCAL:**

Estudo longitudinal realizado em cinco Unidades Básicas de Saúde em Bauru (SP), Brasil.

**MÉTODOS:**

A amostra foi composta de 620 homens e mulheres que foram entrevistados em 2010, 2012 e 2014. O mesmo grupo de pesquisadores realizou as entrevistas utilizando o questionário desenvolvido por Baecke et al. Escores da AF ocupacional, exercícios/esportes, lazer/transporte e AF global foram considerados neste estudo longitudinal. Tendências temporais de AF nos quatro anos de seguimento foram avaliados de acordo com sexo, idade e CE.

**RESULTADOS:**

Verificou-se que, após quatro anos de seguimento, a redução da AF total (-13,6%; intervalo de confiança, IC 95% = -11,9 a -15,3) foi estatisticamente significativa. Além disso, o declínio no domínio ocupacional e no exercício/participação esportiva foram afetados pela idade, enquanto a redução na AF total foi afetada pelo sexo, idade e CE.

**CONCLUSÕES:**

A AF total diminuiu significativamente de 2010 para 2014 em pacientes ambulatoriais do Sistema Único de Saúde, e idade e sexo masculino foram importantes determinantes de AF em seus diferentes domínios.

## INTRODUCTION

Development of new technologies and characteristics within the environment have continued to reduce the amount of energy expenditure on a daily basis.^1^ Physical inactivity is an important risk factor for health, contributing substantially to the worldwide epidemic of non-communicable diseases (NCDs). These cause 5.3 million deaths per year^2^ and lead to significant economic losses in developed and developing countries.^3^^,^^4^^,^^5^^,^^6^^,^^7^

An active lifestyle is important for promoting good health, and monitoring of lifestyles at population level is critical for decision-making regarding public health. Recent data show that, worldwide, one third of adults do not reach the recommended levels of physical activity (PA).^8^ Additionally, data from the Global Burden of Disease (GBD) study published in 2015 found discordant trends for low physical activity according to sex, such that the overall summary exposure value for men increased by 2.4%, whereas the same indicator for women declined by 1.5%.^9^

In Brazil, a national surveillance system was implemented in 2006 to annually collect data on risk factors for non-communicable diseases, including low levels of PA.^10^ This initiative should be recognized as a step forward for public health. However, its limitations, such as the facts that the survey is carried out by telephone, it only involves adults living in the state capital cities and the participants of the sample are not the same every year, need to be acknowledged.

In developing countries, most studies have a cross-sectional design or only assess total or leisure-time PA.^11^^,^^12^^,^^13^ Therefore, they are vulnerable to missing crucial information regarding other PA domains (e.g. occupational, active transportation and sports data). Hence, longitudinal data are important for describing the patterns of habitual PA over time. These make it possible to understand the burden of physical inactivity on health outcomes.^14^

Monitoring of PA within primary care constitutes an important preventive action. This makes it possible to reach a wide portion of the overall population, thereby averting occurrences of diseases relating to physical inactivity and subsequent economic losses.^3^^,^^15^^,^^16^^,^^17^

## OBJECTIVE

The objective of this study was to describe time trends of PA in different domains from 2010 to 2014, among users of the Brazilian National Health System, taking into account the effects of sex, age and economic status (ES).

## METHODS

### Sample

This longitudinal study was conducted from August 2010 to December 2014, in the city of Bauru, which has around 300,000 inhabitants and is located in the state of São Paulo, the most industrialized region of Brazil. Prior to implementation, the study was approved by the Ethics Committee of Universidade Estadual de São Paulo (UNESP), Bauru campus (procedural number 1046/46/01/10), and all participants gave written and verbal informed consent.

The sample size was estimated based on the percentage of the Brazilian population that is covered only by the Brazilian National Health System (60%).^18^ The parameters used in making the estimate were: 3.8% error (arbitrary because there were no other similar studies), 5% statistical significance and 50% design effect. Therefore, at the baseline, a minimum sample size of 960 participants was estimated to be representative, i.e. at least 192 subjects in each primary healthcare unit (PHU) in Bauru.

The primary care of the Brazilian National Health System in Bauru is organized into 17 PHUs, spread out across all geographical regions of the city. To recruit participants for this longitudinal study, we stratified the metropolitan region of the city into five geographical regions in 2010. The biggest PHU of each geographical region was selected to take part in the study. 

The Municipal Health Department provided lists with the names of all patients attended at these PHUs over the preceding six months. Taking these lists into account, 1,915 patients were randomly selected for telephone contact. This overall number of potential participants was estimated considering that there would be one refusal per two subjects invited to take part in the study. 

During the telephone contact, the inclusion criteria were checked. Participants needed to be ≥ 50 years of age and to have lived in the area covered by that specific PHU for at least one year. Patients who fulfilled the inclusion criteria were invited to attend an interview and assessment at their own PHU.

### Physical activity assessment

The same group of researchers (n = 3) conducted the interviews using the questionnaire developed by Baecke et al.,^19^ at the specific BHU, in a quiet room reserved for the study. The version of the questionnaire used in this cohort study had previously been validated for use in Brazilian Portuguese.^20^

The questionnaire comprises 16 questions that are scored on a five-point Likert scale, ranging from never to very often/always. It addresses three domains of PA: occupational, leisure-time/locomotion and exercise/sport participation. The occupational domain is composed of eight questions that take into account behavior adopted during work activities, such as sitting, standing, walking, lifting heavy loads, sweating and feeling tired. The exercise/sport participation domain is composed of one question stratified into three sections that take into account the intensity, weekly duration (in hours) and previous length of practice (in months) of the activities performed by the interviewee. The leisure-time/transportation domain is composed of seven questions that take into account behavior during leisure-time/locomotion, like playing sports, watching television, walking and cycling. The PA level is calculated by means of specific equations and is expressed as scores for each PA domain (higher score denotes higher PA). The sum of all domains constitutes the overall PA.

Changes in PA scores (considering all PA domains) from 2010 to 2014 were calculated and then expressed as z-scores. In the present study, z-scores ≤ -1.5 were treated as significant reductions in PA over the follow-up (dependent variable), and the sample was split up as ≤ -1.5 or > -1.5 for all PA domains.

### Independent variables

The following data were obtained through interviews at the baseline and were structured as categorical variables:

1. Sex (male or female);
2. Age (< 65 years old or ≥ 65 years old); and
3. Economic status (ES; low or middle/high income).

The questionnaire used to estimate ES was a previously validated Brazilian questionnaire,^21^ which specifies the following income groups: low (classes C, D and E, with family income of US$ 76.94-966.38 per month); and middle/high (classes B and A, with family income of US$ 1,823.33-2,703.61 per month at 3.60 currency exchange rate at the time of the study). The questionnaire estimates the income based on data about education attainment, possession of appliances and physical characteristics of the house (e.g. number of toilets).

Additionally, body mass index (BMI) was calculated using measurements of weight and height in each patient and was obtained by dividing weight by squared height (kg/m^2^).

### Statistical analyses

Descriptive statistics were presented as means, medians, standard deviations, interquartile ranges and 95% confidence intervals (95% CI). The effects of sex, age and ES on PA levels were assessed using analysis of variance (ANOVA) for repeated measurements. Mean values were adjusted using the covariance explained by BMI (baseline) and PHU, thus generating estimated means and standard error means. Ordinal data were expressed as percentages, while Cox regression (expressed in terms of hazard ration [HR] and its 95% CI) was used in the multivariate model adopted, to assess associations with ordinal data (reduction of PA was treated as an outcome). All multivariate models generated using Cox regression were simultaneously adjusted according to sex, age, EC, schooling and PHU. All statistical procedures were conducted using the BioEstat software, version 5.2 (BioEstat, Teffe, Amazonas, Brazil) and statistical significance was set at P < 0.05.

## RESULTS

We called each of the 1,915 individuals identified in the PHU lists to invite them for a baseline assessment between August and December 2010; 963 (50.3%) agreed to take part in the longitudinal study. Two years after the baseline assessment (August-December 2012), we approached these subjects again. We were able to trace 802 participants: 161 subjects could not be reached or declined to participate and 25 had died. Finally, between August and December 2014, we attempted to locate all participants again and were able to interview 695 of them: 237 subjects could not be reached or declined to participate and another 34 had died. Taking all three assessment periods into account, we thus had data on 620 participants.

It should be noted that this is an ongoing cohort study, in which the main purpose is to investigate the relationship between healthcare costs at primary care level and behavioral variables (treated as independent variables). During all three years (2010, 2012 and 2014), the medical records of all participants were assessed (n = 904, excluding 59 deaths), but only the participants for whom information about PA was available at all three assessments (2010, 2012 and 2014) were included in this study (n = 620). The final sample was composed of 620 participants (454 women; 73.2%) with the three evaluations and its baseline characteristics according to sex are presented in Table 1.

**Table 1:**
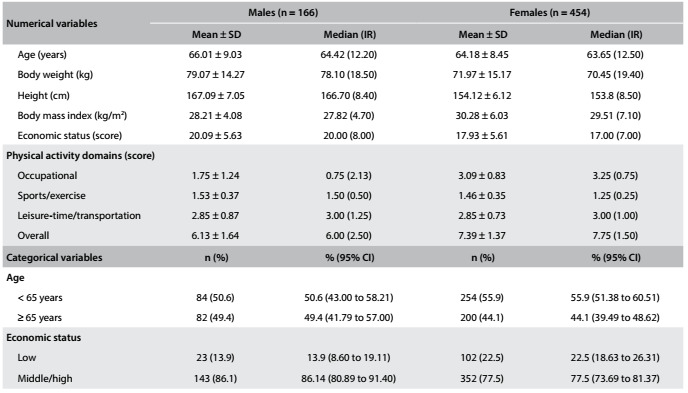
Characteristics of the sample at baseline according to sex (Bauru, SP, Brazil; n = 620)

SD = standard deviation; IR = interquartile range; 95% CI = 95% confidence interval.

Regarding the occupational domain, older people’s PA decreased from 2010 to 2014 (HR = 2.23; 95% CI = 1.29 to 3.86; Table 2). Sex and ES were not associated with modification of occupational PA. Regarding exercise/sports participation, older people presented 90% less likelihood of decreasing their PA in this domain from 2010 to 2014 (HR = 0.10; 95% CI = 0.01 to 0.84; Table 2). On the other hand, sex and ES were not associated with PA modification in this domain. It should be noted that no independent variable was associated with modifications in the leisure-time/transportation domain.

**Table 2:**
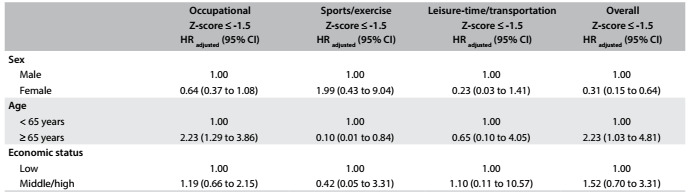
Changes in physical activity from 2010 to 2014 among adults attended through the Brazilian National Health System (n = 620)

HR = hazard ratio; 95% CI = 95% confidence interval; Cox regression simultaneously adjusted for sex, age, economic condition, primary healthcare unit and schooling.

**Figure 1:**
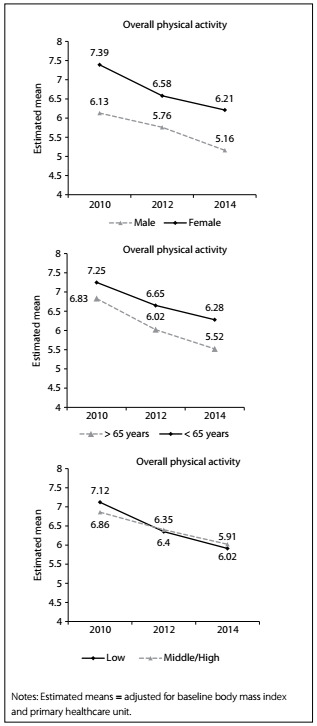
Overall physical activity from 2010 to 2014 according to sex, age and economic status (Bauru, SP, Brazil; n = 620).

Taking into account the overall PA score, women had 69% less chance of decreased PA from 2010 to 2014 (HR = 0.31/ 95% CI = 0.15 to 0.64; Table 2), while older people presented increased likelihood of reduced PA after four years of follow-up (HR = 2.23; 95% CI = 1.03 to 4.81; Table 2). Figure 1 describes the overall PA during the follow-up, according to sex (Figure 1, Panel A), age (Figure 1, Panel B) and ES (Figure 1, Panel C). Overall PA decreased over time independently of the variables (ANOVA parameter “time”), but women (Figure 1A; P-value = 0.001) and younger adults (Figure 1B; P-value = 0.001) were more active than men and older adults. In the overall sample, after four years of follow-up, the reductions in overall PA (-13.6%; -11.9 to -15.3) and leisure-time/transportation PA (-28.3%; -26.1 to -30.5) were statistically significant, while exercise/sports participation increased (13.3%; 10.8 to 15.7) and occupational PA did not decrease significantly (-0.9%; -6.1 to +4.2).

## DISCUSSION

In this four-year longitudinal study, PA among outpatients of the Brazilian National Health System showed a significant decrease over time, mostly among men and older subjects.

Regarding the occupational domain, we observed that older people decreased their PA over the years more than the younger groups, while sex and ES were not associated with significant changes. This finding was expected when considering the age range adopted in this study for the older group, which was higher than the average retirement age in Brazil (55.1 years for men and 52.2 years for women in 2010).^22^ Moreover, in order to encompass retired people who maintain PA at home, in our study we also considered household activities in the occupational domain. In agreement with our findings, recent data show that the largest absolute decline in PA among Brazilians is in the occupational domain, but the largest relative decline is in domestic PA.^1^ The reduction of energy expenditure in this domain is associated with increased overweight/obesity.^23^ Thus, given the growth in prevalence of obesity and its associated morbidity, mortality and economic impact, remaining physically active within the occupational/domestic domain is a key factor for public health policies.

Concerning exercise/sports participation, our results showed that older adults were less likely to decrease their PA in this domain from 2010 to 2014, and no changes according to sex or ES were observed. Considering the age range of the population in our study, it is reasonable to think that the older group (age ≥ 65 years old) was less likely to present modifications to their routines, mainly because most of them had already retired. Moreover, the PA scores in this domain are usually lower in older groups than in younger groups and thus less prone to modifications. Finally, in early old age (65-75 years), there may be a modest increase in physical activity, in an attempt to fill free time resulting from retirement.^24^

Regarding overall PA, women had 69% less chance of decreased PA from 2010 to 2014, while older people presented increased likelihood of reduced PA after four years of follow-up. In this sample, overall PA decreased by 13% during the follow-up period. In general, women spend less time doing exercise/sports activities, but the time spent doing household activities is substantially higher, which increases the overall score in comparison with men (household activities are performed daily, while exercise/sports during leisure time are not). Regarding the decline in overall PA with age, it is well established in the scientific literature that PA decreases from adolescence to adulthood,^25^ and is expected to continue decreasing during the aging process. The declining trend in overall PA usually increases the percentage of body fat and reduces muscle strength, agility, flexibility and endurance, thus compromising the ability to remain physically fit.^26^ However, it is well known that the prevalence and incidence of NCDs increase with age, which emphasizes the importance of becoming physically active or maintaining the existing levels of PA.

The biggest strength of our study consisted of its description of longitudinal patterns of PA in different domains according to sociodemographic characteristics. Thus, considering that one of the biggest public health challenges is to encourage individuals to be physically active, and that lifetime PA contains occupational, domestic, sports, leisure-time and transportation domains, there are many possibilities for people to reach the guideline goals of PA for health.

Nonetheless, greater availability of environments favorable for PA (which includes schools, workplaces, commuting and the built environment) is urgently needed.^27^^,^^28^ Moreover, our findings indicate that development of public policies in Brazil for promoting leisure-time PA for adults and elderly people, particularly to make up for the decline of occupational PA, has not been entirely effective. This means that the amount of money that fails to be spent on public policies to promote PA might be proportional to the increase in healthcare expenditure for this population. If this indicator were to be adjusted, the country could boost its levels of total PA and, consequently, decrease the burden of undesirable health outcomes.

Some limitations should be taken into account in interpreting our results. Firstly, there are no other national studies describing time trends of PA using the same questionnaire, and therefore we were unable to make comparisons. Secondly, PA was self-reported. Although methods of greater accuracy for determining PA levels exist (and these could improve the quality of information), the costs involved and time required to conduct large studies using more direct measurement tools would be greater. Thus, use of a questionnaire seemed more appropriate for this study. Moreover, the same staff members conducted the interviews at all stages of the analysis. Finally, we did not include any information about comorbidities.

## CONCLUSIONS

In summary, overall PA decreased significantly from 2010 to 2014 among these outpatients of the Brazilian National Health System, while age and male sex were important determinants of PA in its different domains. The large decrease in overall PA (more than 10%) is of concern, especially in this specific age group, in which PA has an impact on prevention and treatment of diseases. PA decreased from 2010 to 2014 for people aged under 65 and among and women.
